# Associations Between Relapses and Psychosocial Outcomes in Patients With Schizophrenia in Real-World Settings in the United States

**DOI:** 10.3389/fpsyt.2021.695672

**Published:** 2021-10-26

**Authors:** Dee Lin, Kruti Joshi, Alexander Keenan, Jason Shepherd, Hollie Bailey, Mia Berry, Jack Wright, Sophie Meakin, Carmela Benson, Edward Kim

**Affiliations:** ^1^Janssen Scientific Affairs, LLC, Titusville, NJ, United States; ^2^Janssen Global Services, LLC, Titusville, NJ, United States; ^3^Adelphi Real World, Bollington, United Kingdom

**Keywords:** early intervention, disease burden, symptoms, hospitalisation, psychotic episode

## Abstract

**Aim:** To assess associations between relapses and psychosocial outcomes in adult patients with schizophrenia treated in United States (US) healthcare settings.

**Methods:** Data were derived from a point-in-time survey of psychiatrists and their patients with schizophrenia conducted across the US, France, Spain, China, and Japan between July and October 2019. For the purposes of this analysis, only data from US practitioners and patients were included. Disease-specific programmes (DSPs) are large surveys with a validated methodology conducted in clinical practise; they describe current disease management, disease burden, and associated treatment effects (clinical and physician-perceived). Participating psychiatrists completed patient record forms for their next 10 consecutive adult consulting patients with schizophrenia, with the same patients invited to voluntarily complete a patient self-completion (PSC) questionnaire. Surveys contained questions on the patients' disease background, treatment history, prior hospitalisation due to schizophrenia relapse and a series of psychosocial outcomes. Associations between relapses in the last 12 months and psychosocial outcomes were examined using multiple regression.

**Results:** A total of 124 psychiatrists provided data on 1,204 patients. Of these, 469 patients (mean age, 39.6 years; 56.5% male) had known hospitalisation history for the last 12 months and completed a PSC; 116 (24.7%) patients had ≥1 relapse. Compared to patients without relapses, patients who relapsed were more likely to be homeless, unemployed, previously incarcerated, and currently have difficulties living independently (all *p* < 0.05). Patients who experience a relapse also had greater working impairment and poorer quality of life compared with those who did not relapse. In general, psychosocial outcomes became poorer with an increasing number of relapses.

**Conclusions:** In this population of patients with schizophrenia from the US, relapse was significantly associated with poor psychosocial outcomes, with a greater number of relapses predicting worse outcomes. Early intervention to reduce the risk of relapse may improve psychosocial outcomes in patients with schizophrenia.

## Introduction

Schizophrenia is a chronic and severe mental disorder characterised by abnormal behaviour, distortions of thinking and perception, and impaired daily functioning. It is one of the top 15 health conditions associated with disability worldwide (1) and affects ~1.1% of the adult population in the United States (US) (2). In the US, the annual costs attributable to schizophrenia are $155.7 billion, including $9.3 billion in direct medical costs and $117 billion in indirect costs (3).

Schizophrenia comprises a range of symptoms, referred to as positive symptoms (e.g., delusions and hallucinations) (4), negative symptoms (e.g., reduced emotional expression and avolition) (4), and cognitive symptoms (e.g., disorganised speech, thought, or attention) (5). Most patients develop schizophrenia between the ages of 18 and 35 years (6). Psychiatric comorbidities, including panic disorder, post-traumatic stress disorder, obsessive-compulsive disorder, depression, and substance abuse, are common in patients with schizophrenia (7). This patient population is also at increased risk of other physical conditions such as cardiovascular and metabolic diseases (8).

For most patients, the clinical course is characterised by recurring relapses, which may be due to treatment nonadherence. Relapse often occurs early in the disease course, with ~81.9% of patients experiencing a relapse within 5 years of a first episode of schizophrenia or schizoaffective disorder (9). Relapse is common after treatment discontinuation and longer treatment periods prior to discontinuation do not reduce the risk of relapse. The transition from remission to relapse may be abrupt, with limited early warning signs. After relapses, symptom severity often returns to levels similar to the initial psychotic episode (10). Approximately one in five patients will have poorer long-term outcomes following a relapse (11), and only one in seven will achieve long-term recovery (12). A prospective study that examined the magnetic resonance imaging of brain structures in patients with schizophrenia revealed that relapse duration is associated with a marked decrease in general and regional brain measures (13).

A decline in social functioning is another key consequence of schizophrenia, and it may act as a predictor of poor treatment outcomes (14). Symptomatic treatment in itself is insufficient to restore occupational performance and interpersonal relationships (15). It has been recommended that treatment goals should include not only symptomatic remission but improvements in psychosocial functioning and quality of life (QoL) (16). Accordingly, measurement of social functioning should be considered when assessing the effectiveness of antipsychotic therapy (17). Preventing decline in social function, for instance by providing housing and increasing opportunities for employment, may lead to improved clinical outcomes and reduce risk of relapses.

Schizophrenia relapses may adversely affect psychosocial outcomes such as personal relationships, education, and employment (18) and, consequently, may also lead to poor self-esteem and feelings of hopelessness (19). Patients who experienced relapse have lower Global Assessment of Functioning scores compared with those who did not relapse (20), while multiple relapses may result in greater functional deterioration (21). Patients with a relapsing course of disease are less likely to engage in long-term relationships (22). Repeated relapses are also associated with an increased risk of suicidal behaviour (23).

To date, the associations between schizophrenia relapses and psychosocial outcomes has not been extensively explored using real-word data. The objective of this study was to assess the associations between having relapses and psychosocial outcomes, as well as the incremental association per each additional relapse in adult patients with schizophrenia, by using psychiatrist- and patient-reported survey data collected from US healthcare settings.

## Materials and Methods

### Study Background

Data for this analysis were extracted from the Adelphi Schizophrenia Disease Specific Programme (DSP™), undertaken in the US, France, Spain, China and Japan between July and October 2019. For the purposes of this analysis, only US data were included. DSPs are large surveys with a validated methodology conducted in clinical practise; they describe current disease management, disease burden impact, and associated treatment effects (clinical and physician-perceived). The Adelphi DSP™ methodology has been published (24). The validity of the DSP data has been verified in studies involving comparisons with external data sources and has demonstrated the power of trend data over time (25, 26). This specific DSP is a point-in-time survey of physicians their patients with schizophrenia in a real-world clinical setting.

### Eligible Physicians and Patients

Participating physicians were psychiatrists practising in office- or hospital-based outpatient or inpatient settings at the time of the study, were personally responsible for pharmacological treatment decisions for schizophrenia, and were managing ≥5 patients with schizophrenia in a typical week. Participating patients were aged ≥18 years at the time of data capture, had a psychiatrist-confirmed diagnosis of schizophrenia, and were not currently participating in a clinical trial. To ensure collection of a robust sample of inpatients, the target inpatient/outpatient quota was 20/80%. For this analysis, only patients whose psychiatrist had their full medical history for the last 12 months were included.

### Data Collection

Psychiatrists were asked to identify the next 10 consecutive eligible adult patients with schizophrenia who they consulted in clinical practise and to complete a patient record form (PRF) for each patient. PRFs contain detailed questions on patient demographics, diagnosis, disease management, disease status, symptomatology, tests performed, treatment history, drivers of therapy choice, history of hospitalisation, concomitant conditions, life satisfaction, medication adherence, and impact of the disease on the patient.

Patients who had a PRF completed by their psychiatrists were invited to voluntarily complete a patient self-completion (PSC) questionnaire, which contains detailed questions on their demographic and clinical background, housing circumstances, education, biggest challenges of schizophrenia, disease severity and improvement, social functioning, and medication adherence. As an additional part of the PSC, patients completed validated QoL measures relating to the emotional and physical impact of their condition. Health status was assessed by EuroQol-5 Dimension (EQ-5D) utility score (range 0–1) and Visual Analogue Scale (VAS; range 0–100). For both instruments, lower scores indicate poorer health (27, 28). Overall life satisfaction was assessed by the Quality of Life Enjoyment and Satisfaction Questionnaire (Q-LES-Q-SF) (29), and rated from 1 (very poor) to 5 (very good). Impairment was assessed by the Work Productivity and Activity Impairment (WPAI) scale (30); scores ranged from 0 to 100, with higher scores indicating greater impairment.

PSCs were completed by the patient independently of the psychiatrist immediately after consultation and returned in a sealed envelope to ensure confidentiality. Patients who completed a PSC were grouped according to number of relapses (defined as a schizophrenia-related hospitalisation) in the last 12 months; these data were derived from PRFs. We used relapse-related hospitalisation as the main indicator of relapse to standardise the definition of relapse across patient responders and ensure clinical validation, as hospitalisation can be easily validated by patient medical records.

Psychosocial outcomes reported by patients were categorised as QoL and daily living (i.e., housing situation, getting ready in the morning, EQ-5D utility, Q-LES-Q-SF), social and employment (i.e., family and social interactions, WPAI, difficulties in holding down a job), and emotional effects.

### Ethics

Psychiatrists provided consent to participate and to provide patient information during screening into the survey. Patients completing a PSC provided informed consent for use of their anonymized and aggregated data for research and publication in scientific journals. Data were collected such that patients and psychiatrists could not be identified directly; all data were aggregated and de-identified before receipt.

Data collection was consistent with the European Pharmaceutical Marketing Research Association guidelines (31), and as such ethics committee approval was not required. However, this survey did undergo review by the Western Institutional Review Board and was granted approval. The survey was performed in full accordance with relevant legislation at the time of data collection, including the US Health Insurance Portability and Accountability Act 1996 (32), and the Health Information Technology for Economic and Clinical Health Act legislation (33).

### Statistical Analysis

Patients who completed a PSC were grouped by the number of relapses in the last 12 months (0, 1, 2, and ≥3 relapses). Demographic and clinical characteristics were described for each group using summary statistics. Numeric variables were described using count, mean, standard deviation (SD), median, interquartile range (IQR), min, and max; categorical variables were described using count and percentage. Analysis of variance (ANOVA) and Chi-squared tests were used to compare outcomes across groups for numeric and categorical variables, respectively.

Multiple regression analyses were conducted with selected outcomes as the dependent variable and relapse groups as the main independent variable of interest. Other regression covariates adjusted for were age, sex, body mass index (BMI) and Charlson comorbidity index. The type of regression model varied according to the dependent/outcome variable, with linear regression for numerical outcomes (reported as β coefficients), logistic regression for binary outcomes (reported as odds ratios [ORs]), and multinomial logistic regression where the outcome was categorical with more than 2 categories (reported as relative risk ratios [RRRs]). Wald tests were used to generate *p-*values for each relapse group, with 0 relapse as the reference. Likelihood ratio tests were also used to generate *p*-values comparing 0 relapses against 1, 2, and ≥3 relapses combined (i.e., 0 relapses compared to any number of relapses).

Assumptions for models were checked by examining the residuals for linearity and normality (where appropriate) and examining the variance inflation factors and condition number for multicollinearity. In the majority of models, no issues were observed with either. However, some issues were observed in regressions conducted on a small sample size so these results should be interpreted more cautiously.

All analyses were conducted in Stata v16.1 (StataCorp, 2019).

## Results

A total of 124 office- or hospital-based psychiatrists provided data on 1,204 patients with schizophrenia. A total of 555 patients completed a PSC; of these, 469 patients had known hospitalisation history for the last 12 months and were included in the final analyses.

### Patient Demographics and Clinical Characteristics

Among the 469 patients, 116 (24.7%) were hospitalised due to a schizophrenia relapse in the last 12 months, of which 72 (15.4%), 27 (5.8%), and 17 (3.6%) had 1, 2, or ≥3 relapses, respectively. The mean age for patients with 0, 1, 2, and ≥3 relapses was 40.5, 37.9, 33.4, and 38.4 years, respectively. Most patients were male (0, 1, 2, and ≥3 relapses; 55.8, 54.2, 63.0, and 70.6%) and White (66.9, 62.5, 77.8, and 64.7%). The proportions of patients with a caregiver were 19.0, 36.1, 44.4, and 35.3% (Table 1).

**Table 1:** Patient demographics and characteristics.

		**Number of relapses**				
	**Overall (*n* = 469)**	**0 (*n* = 353)**	**1 (*n* = 72)**	**2 (*n* = 27)**	**≥3 (*n* = 17)**	*P-*value^*^
Age, Mean (SD)	39.6 (15.3)	40.5 (15.6)	37.9 (15.7)	33.4 (10.1)	38.4 (14.5)	0.0808 (AN)
Sex, Male	265 (56.5)	197 (55.8)	39 (54.2)	17 (63.0)	12 (70.6)	0.5600 (CH)
BMI, Mean (SD)	27.0 (4.6)	27.1 (4.5)	26.2 (5.4)	26.6 (3.7)	28.4 (5.0)	0.2906
**Ethnicity**						
White/Caucasian	313 (66.7)	236 (66.9)	45 (62.5)	21 (77.8)	11 (64.7)	0.0794 (CH)
African American/Black	89 (19.0)	65 (18.4)	17 (23.6)	3 (11.1)	4 (23.5)	
Other	67 (14.3)	52 (14.7)	9 (13.9)	3 (11.1)	2 (11.8)	
Has caregiver	111 (23.7)	67 (19.0)	26 (36.1)	12 (44.4)	6 (35.3)	<0.0001 (CH)
Time since diagnosis, years, mean (SD)	9.0 (11.0)	9.0 (11.1)	9.7 (11.1)	5.2 (8.6)	13.3 (11.6)	0.2113 (AN)
**Top 5 comorbidities**						
Anxiety	119 (35.4)	82 (23.2)	21 (29.2)	9 (33.3)	7 (41.2)	0.2079 (CH)
Hypertension	96 (20.5)	78 (22.1)	13 (18.1)	2 (7.4)	3 (17.6)	0.2904 (CH)
Dyslipidemia	73 (15.6)	55 (15.6)	12 (16.7)	3 (11.1)	3 (17.6)	0.9122 (CH)
Depression	67 (14.3)	51 (14.4)	9 (12.5)	3 (11.1)	4 (23.5)	0.6586 (CH)
Obesity	63 (13.4)	47 (13.3)	12 (16.7)	3 (11.1)	1 (5.9)	0.6570 (CH)
No. of comorbidities, mean (SD)	1.5 (1.7)	1.5 (1.7)	1.6 (1.8)	1.4 (1.3)	2.2 (1.8)	0.3342 (AN)
Charlson comorbidity index, Mean (SD)	0.2 (0.7)	0.2 (0.7)	0.2 (0.6)	0.1 (0.5)	0.3 (0.9)	0.6344 (AN)
**Treatment setting**						
Inpatient	53 (11.3)	0 (0.0)	27 (37.5)	16 (59.3)	10 (58.8)	<0.0001 (CH)
Outpatient	416 (88.7)	353 (100.0)	45 (62.5)	11 (40.7)	7 (41.2)	

*Values are n (%) unless indicated otherwise*.

* *Indicates statistical test used*.

*AN, ANOVA; BMI, body mass index; CH, chi-squared; SD, standard deviation; No., Number*.

The mean time since schizophrenia diagnosis for patients with 0, 1, 2, and ≥3 relapses was 9.0, 9.7, 5.2, and 13.3 years, respectively. The most common comorbidity was anxiety (0, 1, 2, and ≥3 relapses; 23.2, 29.2, 33.3, and 41.2%), followed by hypertension (22.1, 18.1, 7.4, and 17.6%). The mean number of comorbidities was 1.5, 1.6, 1.4, and 2.2, respectively. The proportions of patients treated as outpatients were 100.0, 62.5, 40.7, and 41.2% (Table 1).

### Associations Between Schizophrenia Relapses and Psychosocial Outcomes in the Last 12 Months

#### Daily Living and Quality of Life

**Figure 1:**
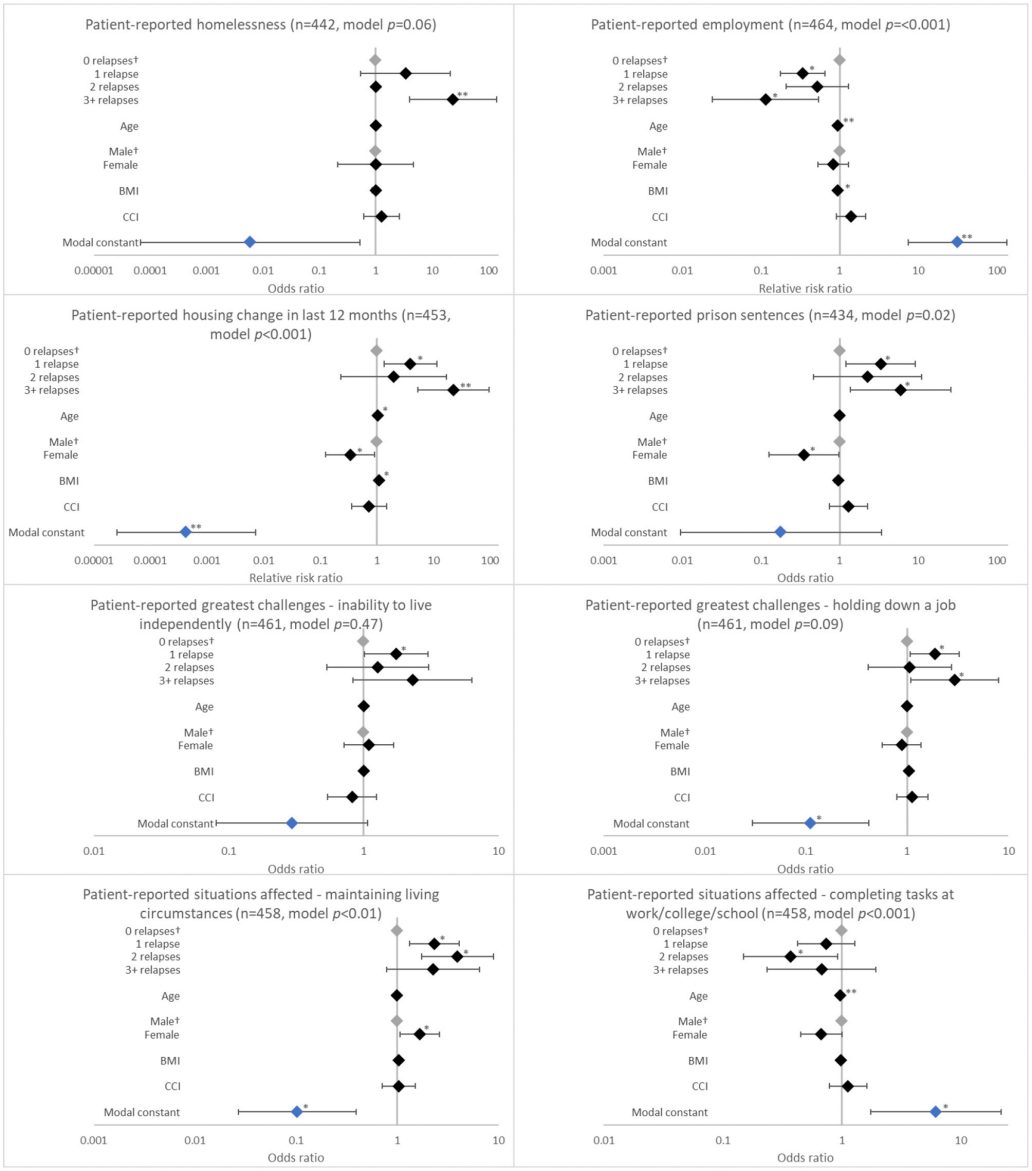
Association between patient-reported psychosocial outcomes and the number of relapses in the last 12 months. ^†^Base level, *p < 0.05 and **p < 0.001. Likelihood ratio *p*-values are referenced in Table 2. BMI, body mass index; CCI, Charlson comorbidity index. Grey diamonds indicate base level. Blue diamond indicates the model constant.

Analysis showed an association between the number of relapses in the last 12 months and having had a housing change in the last 12 months (Table 2, *p* = 0.0018). The likelihood of a negative housing change such as becoming homeless due to their schizophrenia increased more than three-fold with 1 relapse (adjusted RRR 3.932; *p* = 0.013) and was greatest for patients with ≥3 relapses (adjusted RRR 22.334; *p* < 0.001) when compared to patients with no relapses (Figure 1). Number of relapses was also associated with having served a prison sentence (*p* = 0.0067) (Table 2). The likelihood of having served a prison sentence increased more than three-fold with 1 relapse (adjusted OR 3.329; *p* = 0.021) and was greatest with ≥3 relapses (adjusted OR 5.976; *p* = 0.018) when compared with no relapses (Figure 1).

**Table 2:** Association of number of relapses and psychosocial outcomes.

**Outcome (*n*)**	**Likelihood ratio**
Homelessness (*n* = 442)	*p* = 0.0237
Housing change in last 12 months (*n* = 453)	*p* = 0.0018
Served prison sentence (*n* = 432)	*p* = 0.0067
**EQ-5D**	
Utility (*n* = 454)	*p* < 0.0001
VAS (*n* = 460)	*p* = 0.0033
Q-LES-Q-SF (*n* = 359)	*p* = 0.0003
Inability to live independently (*n* = 461)	*p* = 0.0244
Getting ready in the morning (*n* = 458)	*p* = 0.0172
Completing tasks at work/college/school (*n* = 458)	*p* = 0.0423
Completing tasks/chores at home (*n* = 458)	*p* = 0.1328
Maintaining living circumstances (*n* = 458)	*p* = 0.0001
Mealtimes (*n* = 458)	*p* = 0.1829
Maintaining an interest in things (*n* = 458)	*p* = 0.0490
No activities or situations affected (*n* = 458)	*p* = 0.5476
Employment (*n* = 464)	*p* = 0.0001
**WPAI**	
Work time missed (*n* = 141)	*p* < 0.0001
Work impairment (*n* = 152)	*p* = 0.0252
Overall impairment (*n* = 136)	*p* = 0.0048
Activity impairment (*n* = 433)	*p* < 0.0001
Holding down a job (*n* = 461)	*p* = 0.0171
Relationships at work/college school (*n* = 458)	*p* = 0.7958
Personal reputation (*n* = 458)	*p* = 0.8375
Building trust/relationships/social interactions (*n* = 461)	*p* = 0.3256
Family/social interactions (*n* = 458)	*p* = 0.0025
**Emotional effects**	
Embarrassed (*n* = 459)	*p* = 0.0024
Lonely (*n* = 459)	*p* = 0.0032
Scared (*n* = 459)	*p* = 0.0001
Angry (*n* = 459)	*p* = 0.0211
Defeated (*n* = 459)	*p* = 0.0048
Upset (*n* = 459)	*p* = 0.1799
Hopeless (*n* = 459)	*p* = 0.0090
Confused (*n* = 459)	*p* = 0.0038

*EQ-5D, EuroQol 5-Dimension; Q-LES-Q-SF, Quality of Life Enjoyment and Satisfaction Questionnaire; VAS, Visual Analogue Scale; WPAI, Work Productivity and Activity Impairment Scale*.

**Figure 2:**
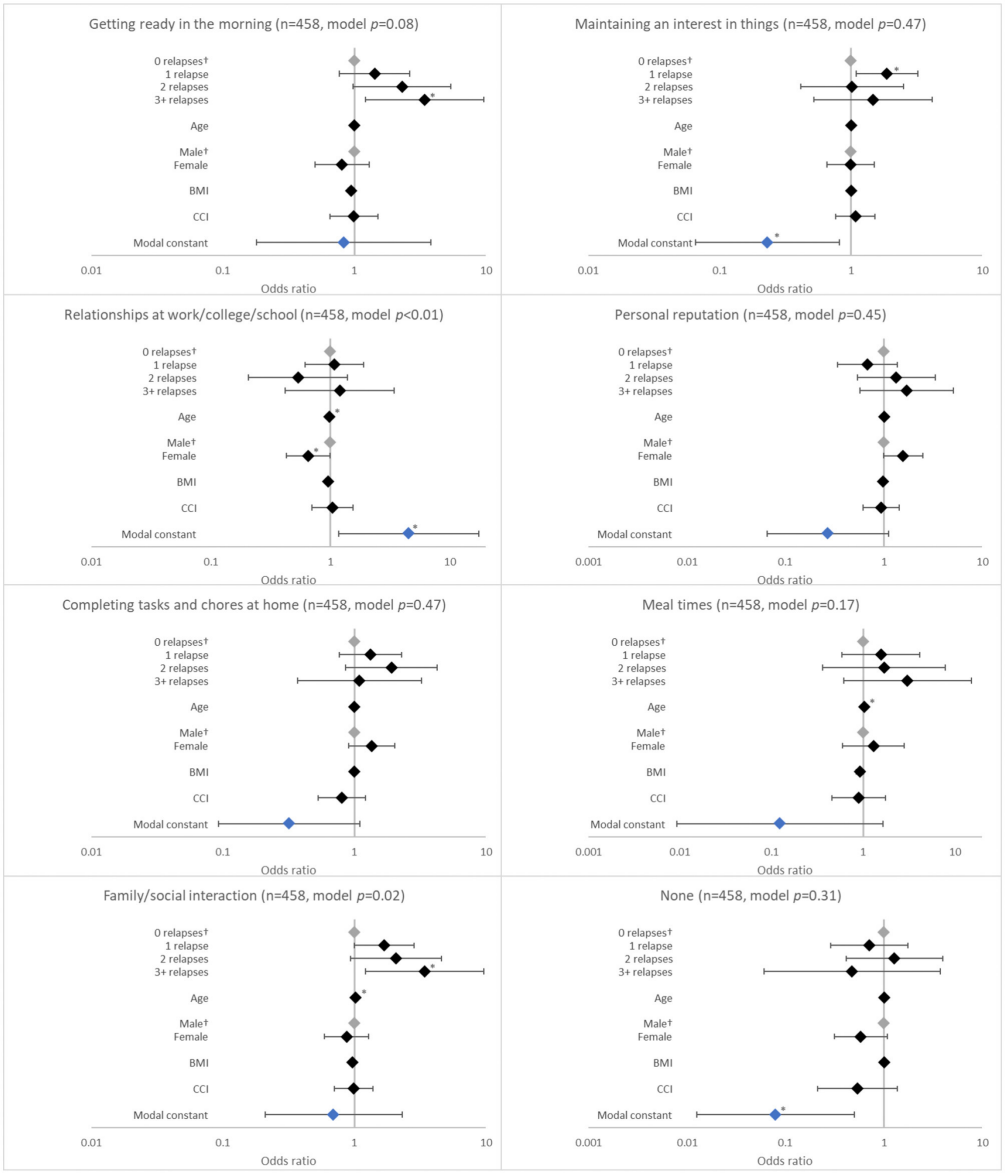
Association between patient-reported social outcomes and the number of relapses in the last 12 months. ^†^Base level, *p < 0.05 and **p < 0.001. Likelihood ratio *p*-values are referenced in Table 2. BMI, body mass index; CCI, Charlson comorbidity index. Grey diamonds indicate base level. Blue diamond indicates the model constant.

Relapse in the last 12 months was associated with having schizophrenia affect getting ready in the morning (*p* = 0.0172). The likelihood of having schizophrenia affect getting ready in the morning increased with number of relapses, although significance was observed only with ≥3 relapses (adjusted OR 3.422; *p* = 0.020; Figure 2).

**Figure 3:**
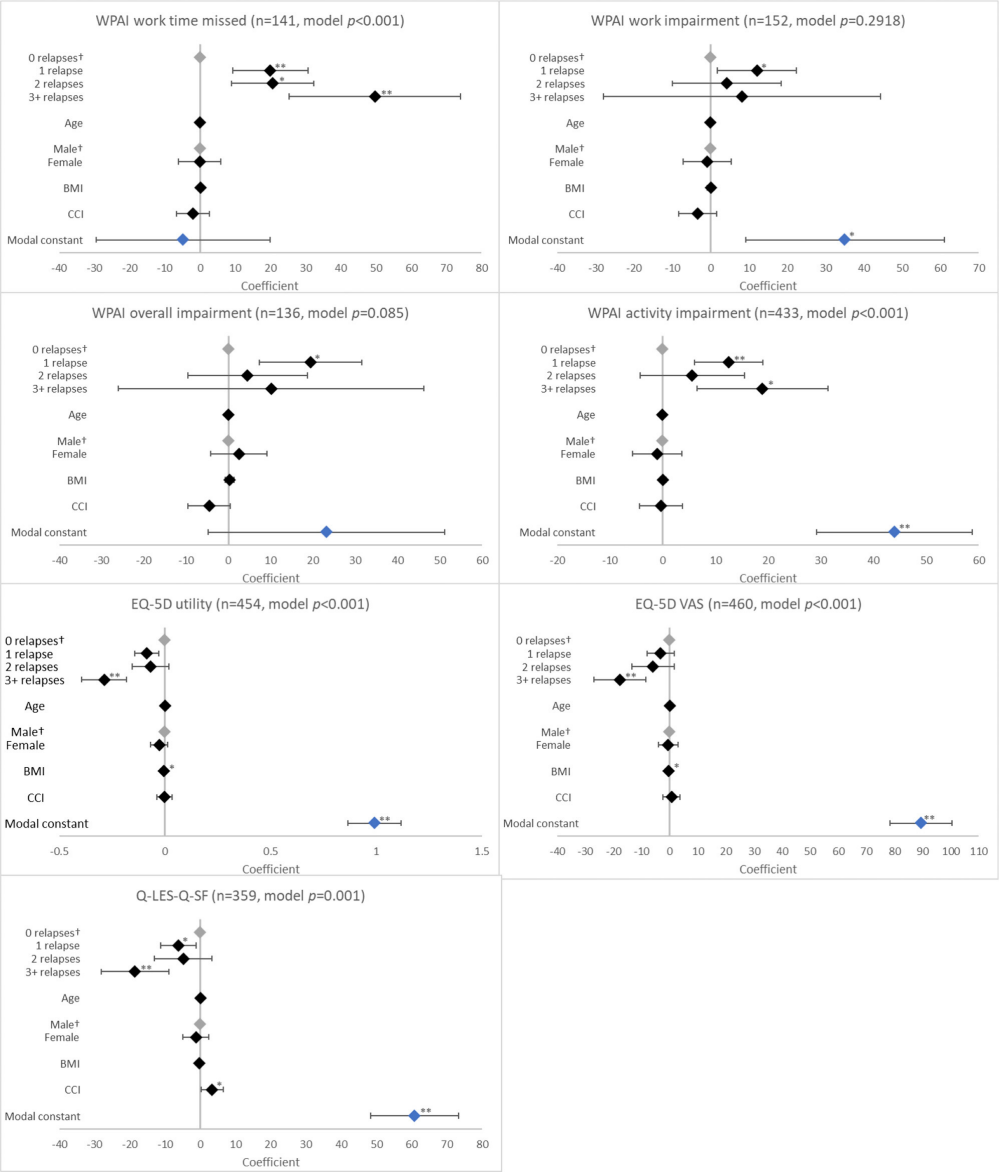
Association between validated patient-reported outcomes and the number of relapses in the last 12 months. ^†^Base level, *p < 0.05 and **p < 0.001. Likelihood ratio *p*-values are referenced in Table 2. BMI, body mass index; CCI, Charlson comorbidity index; EQ-5D, EuroQol 5-Dimension; Q-LES-Q-SF, Quality of Life Enjoyment and Satisfaction Questionnaire; VAS, Visual Analogue Scale; WPAI, Work Productivity and Activity Impairment Scale. Grey diamonds indicate base level. Blue diamond indicates the model constant.

With regard to health status and QoL, there was an association between having a relapse in the last 12 months and EQ-5D utility (*p* < 0.0001), EQ-5D VAS (*p* = 0.0033), and Q-LES-Q-SF (*p* = 0.0003) scores. When compared to no relapses, significantly reduced scores were reported for patients with 1 relapse (EQ-5D utility, coefficient [β] −0.086, *p* = 0.003; EQ-5D VAS, β −3.256, *p* = 0.186; Q-LES-Q-SF, β −6.221, *p* = 0.016); the greatest reduction in these scores was observed with ≥3 relapses (EQ-5D utility, β −0.288, *p* < 0.001; EQ-5D VAS, β −17.805, *p* < 0.001; Q-LES-Q-SF, β −18.527, *p* < 0.001; Figure 3).

#### Social Life and Employment

Relapses in the last 12 months were negatively associated with employment (*p* = 0.0001). Patients who experienced 1 relapse in this period were only 33.8% (adjusted RRR 0.338; *p* = 0.001) as likely to be employed when compared with patients with no relapses; this value decreased to 11.4% (adjusted RRR 0.114; *p* = 0.006) with ≥3 relapses (Figure 1).

Relapses in the last 12 months were also associated with challenges holding down a job (*p* = 0.0171) and with family/social interactions (*p* = 0.0025). The likelihood of having challenges holding down a job increased with 1 relapse (adjusted OR 1.879; *p* = 0.027) and was greatest with ≥3 relapses (adjusted OR 2.943; *p* = 0.035) when compared with no relapses. The likelihood of experiencing challenges with family/social interactions increased with number of relapses, although significance was observed only with ≥3 relapses (adjusted OR 3.434; *p* = 0.020) (Figure 1).

Relapses in the last 12 months were also associated with work time missed and activity impairment according to WPAI (both *p* < 0.0001). Work time missed increased with number of relapses (β all *p* < 0.01). For activity impairment, patients with ≥3 relapses (β = 18.933; *p* = 0.003) reported greater impairment than those with 1 relapse (β = 12.476; *p* < 0.001; Figure 3).

#### Emotional Effects

**Figure 4:**
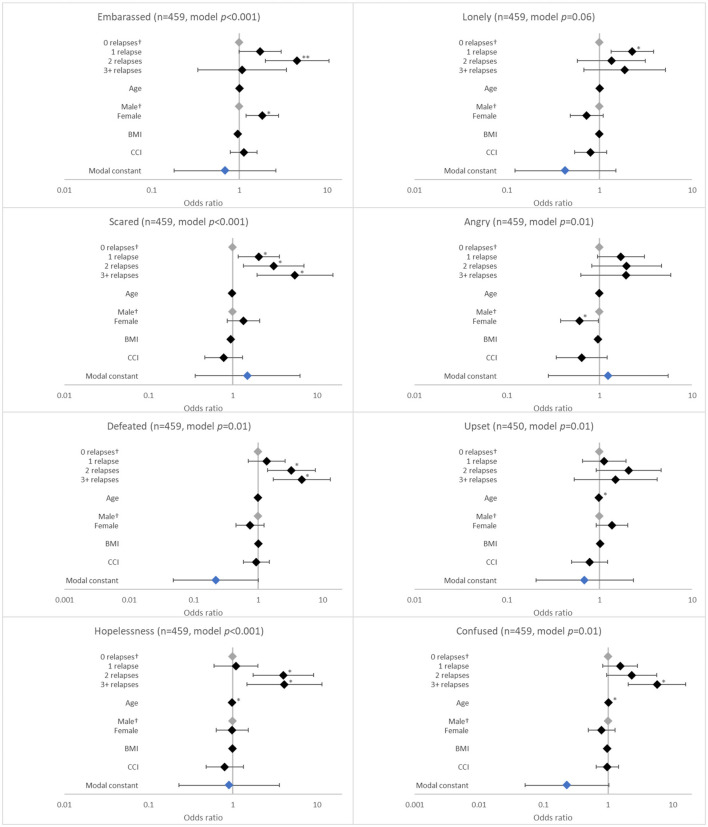
Association between patient-reported emotions and the number of relapses in the last 12 months. ^†^Base level, *p < 0.05 and **p < 0.001. Likelihood ratio *p*-values are referenced in Table 2. BMI, body mass index; CCI, Charlson comorbidity index. Grey diamonds indicate base level. Blue diamond indicates the model constant.

The number of relapses in the last 12 months was associated with several negative emotional effects. These included feeling scared (*p* < 0.001), defeated (*p* = 0.005), hopeless (*p* = 0.009), and confused (*p* = *0.0*04). The likelihood of experiencing these emotions increased with number of relapses, although the ORs were significant for each relapse category only for feeling scared (all *p* < 0.05; Figure 4).

## Discussion

In this population of patients with schizophrenia from the US, we found that schizophrenia relapse was associated with poor psychosocial outcomes and reduced QoL, with these outcomes being worse in patients who experienced a greater number of relapses in the last 12 months. In particular, we observed that patients with more frequent relapses had a higher likelihood of self-reported negative emotional effects, such as feeling defeated or hopeless. Prior research has shown that feelings such as these may influence suicide risk in patients with schizophrenia. One report that reviewed 27 studies of suicide risk in schizophrenia revealed that demoralisation was prevalent in patients with schizophrenia and that depression and suicide were moderated by hopelessness (34). Another study reported that patients with schizophrenia and concurrent depression had poorer long-term functional outcomes compared with those that did not experience depression (35). Evidence suggests that patient education and encouraging their active participation in treatment could play a key role in lessening feelings of hopelessness, as it may give patients an increased sense of control over their disease (36), which may prevent depression associated with lack of control (37).

When compared with patients with no relapses, poorer psychosocial outcomes were observed even after a single relapse. Furthermore, as relapses increased, patients were more likely to experience challenges in normal daily activities, such as difficulty getting ready in the morning. These findings were consistent with previous research, which suggested that repeated relapses may lead to increased social disability (38). An inability to perform normal daily tasks is likely to cause further distress to these patients, as a stable daily routine has been linked to feelings of wellbeing across the general population (39). This may be true to an even greater extent in those with schizophrenia (40). These findings suggest that reducing relapse rates will be a key factor in enabling patients to continue their daily routine, which may have further benefits for their psychological well-being.

Our results also suggest that relapse was associated with reduced employment, increased absenteeism, and reduced work productivity. This was similar to findings from previous research, which reported that reduced relapse rates could significantly improve patients' ability to remain employed (41, 42). Work productivity impacts not only the patient individually, but also has indirect costs to society. A US-based study determined that the economic burden of schizophrenia in 2013 was ~$155.7 billion, of which 38% was due to excess costs associated with unemployment (3). There is some evidence that therapies such as cognitive rehabilitation training (41) and increased time spent in the community (43) could reduce symptom levels and rates of relapse and, thus, decrease unemployment in patients with schizophrenia.

Overall, our findings showed that prevention of relapse could greatly improve patients' lives, with the best way to achieve this being early intervention. Studies have shown that early diagnosis and treatment could help to protect against the progressive structural abnormalities that begin developing in the brain early in the course of schizophrenia (44), and it has been posited that intervention at the prodromal phase of disease may improve long-term outcomes in schizophrenia (45).

The multi factorial nature of schizophrenia means that it will likely require integrated treatments, with both cognitive interventions and pharmacological treatments being used alongside each other to improve many of the psychosocial outcomes we found to be associated with greater relapse number in these patients. For example, using antipsychotic medications to treat the underlying abnormalities in neurotransmitter concentrations alongside psychosocial interventions that can equip patients with the tools needed to deal with real-world situational stressors when they arise (45). If such treatment could be provided, it would allow them to avoid hospital admission while still getting help managing their acute psychotic episodes (46). There is also evidence that treatment may be more effective if provided by multi disciplinary community teams as opposed to more traditional hospital-based services, as patients tend to find that more of their needs were met and they were more satisfied with the care received from multi-disciplinary teams. It has been postulated that this is due to targeting of resources more efficiently to those that required them by multi disciplinary teams compared with more traditional services (47).

In this study, we used relapse-related hospitalisation as a proxy for relapse as it allowed us to both standardise the definition of relapse across patients and it could be validated using patient medical records. To ensure that these hospitalizations accurately reflected relapse rates, we included only hospitalizations that resulted from a relapse of schizophrenia in these analyses and no other types of hospitalizations. Although there are currently no generally accepted criteria for defining relapse in schizophrenia (44), our rationale for using this method is supported by previous studies that have also used this approach (48–50).

The method we used to collect data means that the causality of the association between relapse and psychosocial outcomes could not be definitively concluded; therefore, it is possible that poor psychosocial outcomes may have led to more frequent relapses rather than relapses causing poorer psychosocial outcomes. Other research has confirmed this possibility, with 2 literature reviews reporting that psychosocial intervention reduced relapse in patients with schizophrenia by improving patient general well-being and adherence to medications (51, 52). Further research is needed to assess causality of relapse and psychosocial outcomes in order to better understand their relationship. For example, a controlled trial could compare patients undergoing psychosocial intervention vs. those who do not, with both groups stratified by the same number of relapses.

This study has several limitations. This was an observational, point-in-time study and was based on convenience sampling. Data were collected in a non-randomised approach that depended on consecutive collection of patients that may or may not constitute a representative sample. The data collected and analysed for this study were from US patients and consulting psychiatrists who may see a selective group of patients, such as those who consult more regularly; hence, this sample may not be representative of the wider population of patients with schizophrenia. However, in this analysis, the inclusion and exclusion criteria was minimal, which favored a board sample population. Patients were assigned based on number of relapses in the past 12 months. This non-random assignment may lead to imbalances in risk factors between the groups being compared and, thus, bias the estimates of the effect of number of relapses. Propensity score matching could have addressed this bias but was not performed in this study. For psychiatrists, the inclusion criteria were based on the necessity of participating psychiatrists to see a minimum volume of patients to allow them to complete all survey components in the timeframe allocated and to be actively involved in patient treatment decisions. However, channelling bias may have been introduced in the selection of psychiatrists. Although the point-in-time study design prevents any conclusions about causal relationships, identification of associations is possible. Recall bias may also have affected patient and psychiatrist responses to the questionnaires, which is a common limitation of surveys. However, the data were collected at the time of each patient's consultation and psychiatrists had access to the patient's medical history, both of which are expected to reduce the likelihood of recall bias. As patient completion of these questionnaires was voluntary, the number of responses was inevitably lower than PRF completion. Some base sizes for certain patient subpopulations were small, and results should be interpreted with caution.

## Conclusions

This US-based, point-in-time survey revealed poor psychosocial outcomes in patients with schizophrenia who experienced a relapse in the past 12 months, with poorer outcomes linked to higher numbers of relapses. Patients who had fewer relapses experienced better QoL, greater participation in relationships, lower symptom levels, and better levels of employment compared with patients who experienced more frequent relapses. These results suggest that using appropriate interventions to reduce the risk of relapse is critical to preserving psychosocial function and improving clinical outcomes in people living with schizophrenia.

## Data Availability Statement

The datasets presented in this article are not readily available because all data that support the findings of this study are the intellectual property of Adelphi Real World. Requests to access the datasets should be directed to jason.shepherd@adelphigroup.com.

## Ethics Statement

The studies involving human participants were reviewed and approved by Western Institutional Review Board. The patients/participants provided their written informed consent to participate in this study.

## Author Contributions

DL, CB, AK, JS, HB, MB, and JW contributed to conception and design of the study. SM performed the statistical analysis. All authors contributed to manuscript revision, read, and approved the submitted version.

## Funding

Data collection was undertaken by Adelphi Real World as part of an independent survey, entitled the Adelphi Schizophrenia Disease Specific Programme, sponsored by multiple pharmaceutical companies of which one was Janssen Pharmaceuticals. Janssen Pharmaceuticals did not influence the original survey through either contribution to the design of questionnaires or data collection. The study described here using data from the Adelphi Schizophrenia Disease Specific Programme was funded by Janssen Pharmaceuticals. Medical writing support under the guidance of the authors was provided by Derek Ho, ScriboMedica Ltd., on behalf of Adelphi Real World and Janssen Pharmaceuticals, and was funded by Janssen Pharmaceuticals in accordance with Good Publication Practise (GPP3) guidelines.

## Conflict of Interest

DL, KJ, AK, and CB are employees of Janssen Pharmaceuticals, and hold stock in Johnson and Johnson. EK is currently an employee of Biohaven Pharmaceuticals and was an employee of Janssen Pharmaceuticals at the time of the study. The remaining authors declare that the research was conducted in the absence of any commercial or financial relationships that could be construed as a potential conflict of interest.

## Publisher's Note

All claims expressed in this article are solely those of the authors and do not necessarily represent those of their affiliated organizations, or those of the publisher, the editors and the reviewers. Any product that may be evaluated in this article, or claim that may be made by its manufacturer, is not guaranteed or endorsed by the publisher.
